# A Novel Strain D5 Isolated from *Acacia confusa*

**DOI:** 10.1371/journal.pone.0049236

**Published:** 2012-11-15

**Authors:** Baoling Huang, Chengqun Lv, Yili Zhao, Rong Huang

**Affiliations:** Forestry College, Guangxi University, Nanning, China; Centro de Investigación y de Estudios Avanzados del IPN, Mexico

## Abstract

We isolated a novel strain D5 from nodules of *Acacia confusa*. Under strict sterile conditions the strain could successfully nodulate *Acacia confusa*, *A. crassicarpa* and *A. mangium*, with nitrogenase activity ranging from 18.90 to 19.86 nmol·g^−1^·min^−1^. In the phylogenetic tree based on a complete 16S rRNA gene sequence, the sequence of strain D5 shared 99% homology with that of four species of genus *Pseudomonas*. The 685 bp *nodA* fragment amplified from strain D5 shared 95% homology with the *nodA* sequence of 9 species of genus *Bradyrhizobium*, with a genetic distance of 0.01682. The 740 bp *nifH* gene fragment was amplified from strain D5. This strain D5 *nifH* gene and *Bradyrhizobium* spp. formed a branch, showing 98% homology and a genetic distance of 0. The homology between this branch and the *Bradyrhizobium* spp. DG in another branch was 99%, with a genetic distance of 0.007906. These results indicate that this strain D5 is a new type of nitrogen-fixing bacterium.

## Introduction

In the recently years, many new genera and species of symbiotic nitrogen-fixing microorganisms of legumes have been discovered using emerging new technology [1]. Currently 56 species, 11 genera symbiotic nitrogen-fixing bacteria have been identified, including not only traditional rhizobia that belong to genera *Rhizobium*, *Sinorhizobium*, *Mesorhizobium*, *Bradyrhizobium*, *Azorhizobium* and *Agrobacterium*, but also some symbiotic nitrogen-fixing bacteria that have previously been categorized as non-symbiotic: some species in *Phyllobacterium*, *Ochrobacterum*, and *Methylobacterium* can all grow symbiotically with legumes and fix nitrogen [2]–[6]. The discovery of these “non-traditional” rhizobia has greatly enriched the studies on nitrogen-fixing symbiosis.

*Acacia* is a generic name for all the arbor plants in subfamily Mimosoideae, genus *Acacia*. So far, Rhizobia strains have been successfully isolated and purified from the root nodules of species including *A. melanoxylon*, *A. impdexa*, *A. dealbata*, *A. mearnsii*, *A. auriculiformis*, *A. mangium*, *A. crassccarp*, *A. mangium*×*A. auriculiformis*, *A. confusa*, and *A. cincinnata*, and their physiological, biochemical properties and stress resistance have been studied [7]–[14], showing a diversity of symbiotic Rhizobia of *A.* spp.

Lajudie [15] examined the rhizobia isolated from *Acacia* in Senegal of the Sudan and found that they belonged to *Sinorhizobium* and *Mesorhizobium*, respectively. Zhang et al. [16] characterized 115 phenotypes of 60 rhizobia strains in genus *A. senegal* and found that at a mean genetic distance of 0.725 (which seperates *Rhizobium* and *Bradyrhizobium*) these rhizobia could be divided into 19 strains. Trinick [17]–[19] reported that rhizobia isolated from *Acacia* are of fast-growing type; some strains were close to fast-growing *Rhizobium meliloti*. Lv et al. [20] sequenced and analyzed the complete 16S rDNA and the *nifA* gene fragment of Rhizobia strain HJ06 from *A. crassicarpa*; they showed that in the phylogenetic tree constructed with the 16S rDNA sequence of strain HJ06, strain HJ06 belonged to a *Rhizobium* branch, and the cloned *nifA* gene fragment shared 99.3% homology with *Klebsiella pneumoniae*. Huang et al. [21] performed comparative cluster analysis on the complete 16S rDNA sequence of strain JJ06 from *A. cincinnata*, and found that this strain was phylogenetically located on the *Mesorhizobium* branch. Wang et al. [22] conducted a phylogenetic study on 9 strains of rhizobia isolated from *Acacia* using partial sequences of two housekeeping genes *atpD* and *glnII*, and compared the result with the phylogenetic tree constructed with 16S rDNA gene sequence; the results showed that Rhizobia isolated from *A. mangium* in Fujian and Guangdong belonged to *Mesorhizobium*, whereas strain isolated from *A. confusa* in Guangdong belonged to *Bradyrhizobium*.

Guangxi is located in the north tropical and Subtropical area, the geological condition of which supports life greatly. Thus biodiversity in this region is massive, including that of the microorganisms in the soil. As alien species, *A.* spp. may exchange information including genetic information with local species in addition to adapting to the local environment when cultivated in Guangxi. In particular, gene exchange is extremely likely to happen among strains in species such as *Acacia* rhizobia that show relatively large phylogenetic variations. Aside from transitional *Acacia* rhizobia, are there endophytic bacteria that are “non-traditional Rhizobia” but are symbiotic with *Acacia* plant and can nodulate to fix nitrogen? In this study, a “non-traditional” rhizobia, strain D5 isolated from *A. confusa* was investigated in depth. Inoculation and cross inoculation of this strain were performed under strict sterile conditions, and the nitrogenase activities of its nodules were measured; the complete 16S rRNA sequence of strain D5 and its *nodA* and *nifH* gene sequences were examined for symbiotic nodulation genes and nitrogen-fixing genes, and the phylogenetic position of this strain and the homology of its *nodA* and *nifH* genes were analyzed. Our findings are of great significance in accurately revealing the diversity and the phylogenetic position of *Acacia* rhizobia and in searching for potential lateral gene transfer and gene recombination, thus provides a scientific basis for researches that truly uncover phylogenetic relationships in biological systems.

**Figure 1:**
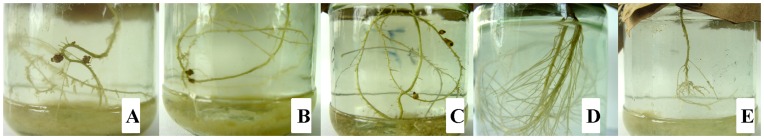
Nodulation of strain D5 inoculated plants. A. *Acacia confusa*, B. *Acacia crassicarpa*, C. *Acacia mangium*, D. *Glycine max,* and E. Control.

## Results

### Inoculation and Cross-inoculation of Strain D5 and Nitrogenase Activity

Under strict sterile conditions, brown spherical nodules were observed 90 d after strain D5 was nodulated to *A. confusa* (**Fig. 1A**), *A. crassccarp* (**Fig. 1B**) and *A. mangium* (**Fig. 1C**), and all these nodules exhibited nitrogenase activity (**Table 1**). No nodules were observed in inoculated *Glycine max* (Linn.) Merr. (**Fig. 1D** **,** **Table 1**) and in the control plants (**Fig. 1E**, **Table 1**) that were not inoculated or inoculated with other *Pseudomonas* strains.

**Table 1:** The nodulation rates of strain D5 inoculated *Acacia confusa*, *A. crassicarpa, A. mangium*, and *Glycine max*, and the corresponding nitrogenase activities of root nodules.

Inoculated plant	Nodule number (#·plant^−1^)	Nodule weight (mg·plant^−1^)	Nodulation rate (%)	Nitrogenase activity (nmol·g^−1^·min^−1^)
*Acacia confusa*	2.76	8.0	75.8	19.86
*Acacia crassicarpa*	2.58	7.0	74.8	18.90
*Acacia mangium*	2.61	7.5	74.9	19.01
*Glycine Max*	0	0	0	0
Control	0	0	0	0

### The 16S rRNA Sequence of Strain D5 and the its Phylogenetic Analysis

**Figure 2:**
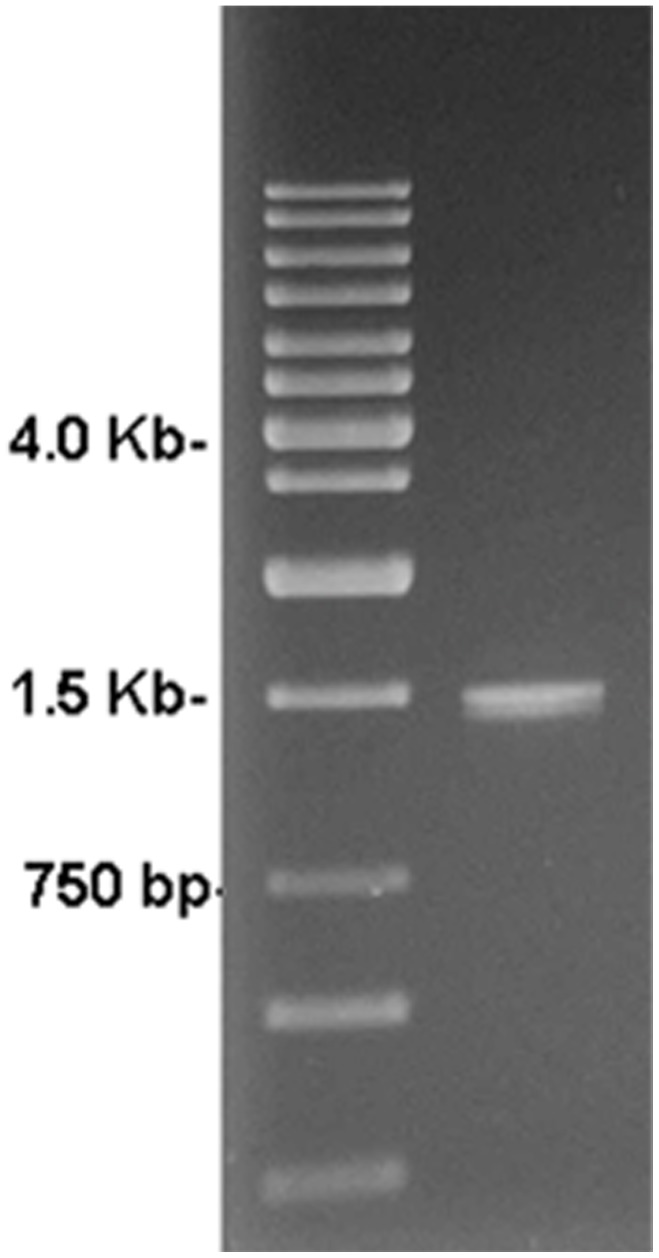
PCR results of 16S rRNA of strain D5.

The PCR amplification product of strain D5 was around 1.5 kb as detected from the agarose gel electrophoresis (**Figure 2**). The completed sequence was 1470 bp. We submitted this sequence to the GeneBank and obtained a record number as D5.sqn 16S_rRNA JX162029.

**Figure 3:**
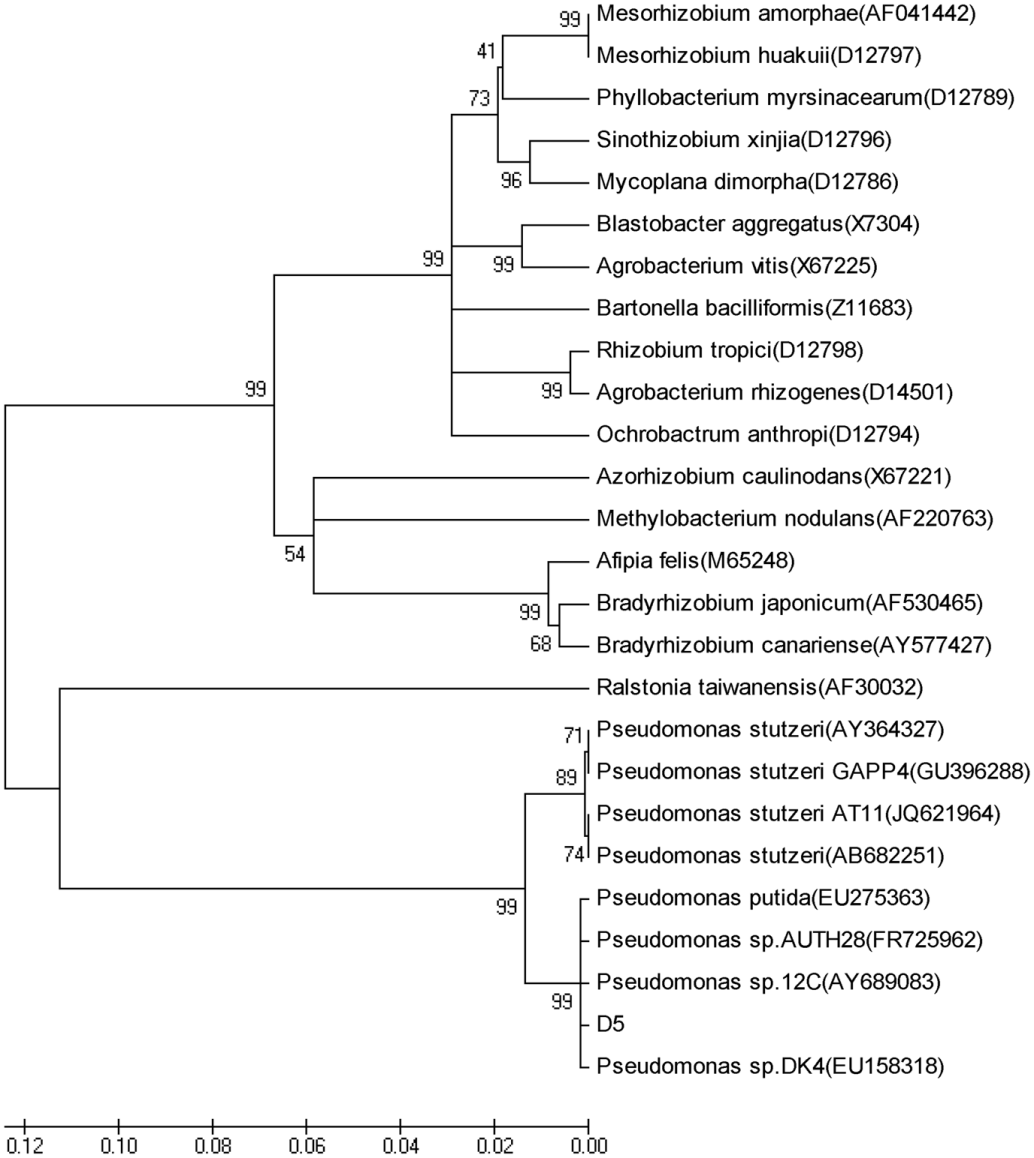
Phylogenetic dendrogram of strain D5 based on 16S rRNA gene sequence.

**Figure 4:**
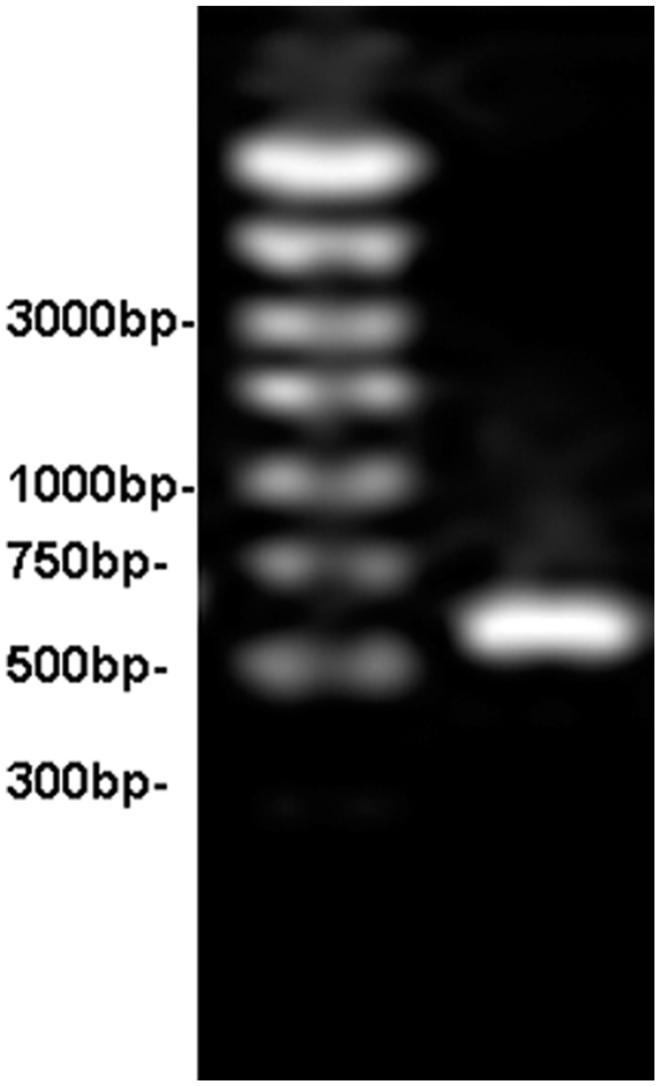
PCR results of strain D5 *nodA* gene.

**Figure 5:**
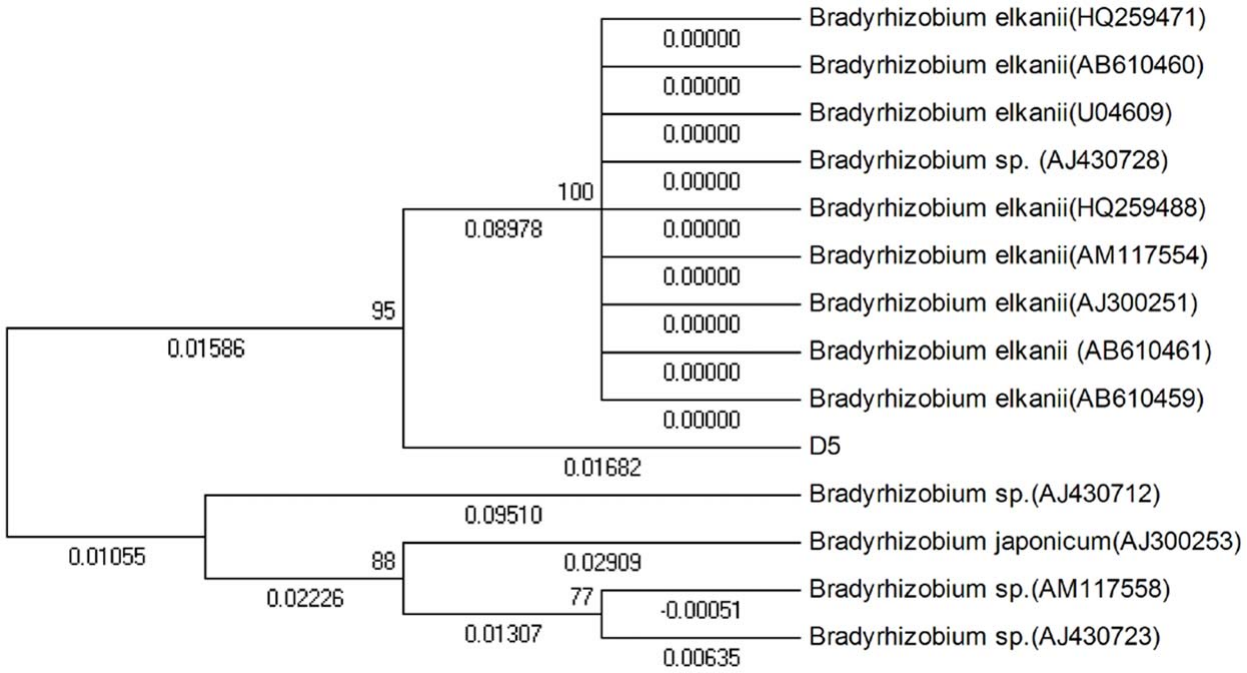
Phylogenetic dendrogram of strain D5 *nodA* sequence.

**Figure 6:**
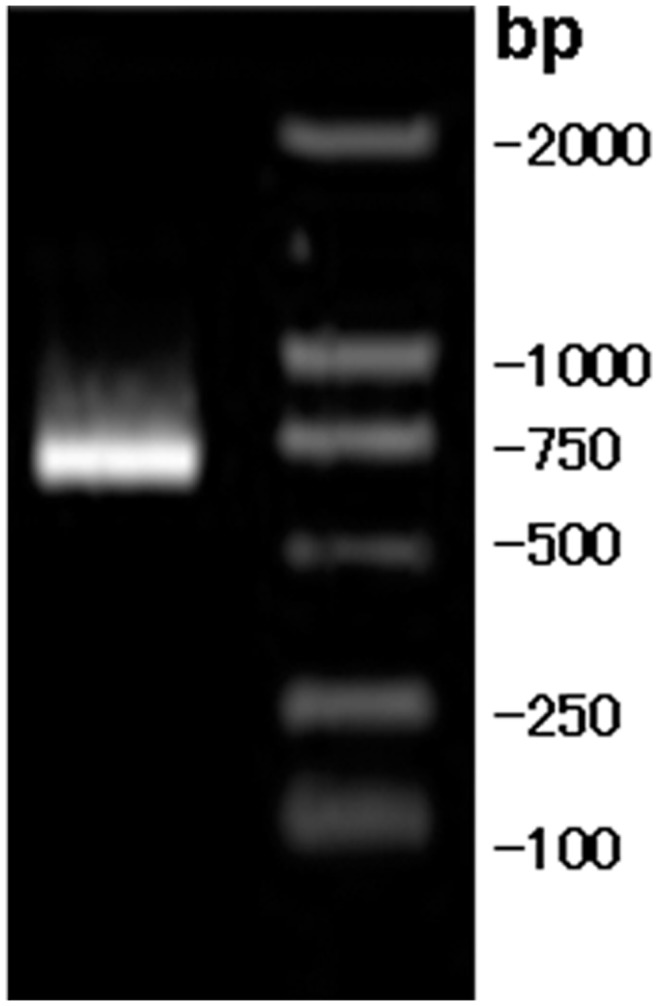
PCR results of strain D5 *nifH* gene.

**Figure 7:**
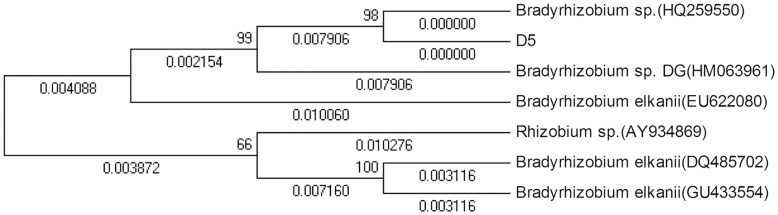
Phylogenetic dendrogram of strain D5 *nifH* gene.

As seen from the phylogenetic tree based on the16S rRNA sequence, strain D5 and 8 strains of *Pseudomonas* were in the same branch. Strain D5 had a genetic distance very close to *Pseudomonas putida* (EU275363), *P.* sp. DK4 (EU158318), *P.* sp. AUTH 28 (FR725962) and *P.* sp. 12C (AY689083). The homology was 99%. Strain D5 also had a very close distance to *P. stutzeri* (AY364327), *P. stutzeri* GAPP4 (GU396288), *P. stutzeri* AT11 (JQ621964), and *P. stutzeri* (AB682251), while traditional rhizobia were located in another branch, far from genus of *Pseudomonas* (**Figure 3**). As the criterion for diving plants into species using the 16S rRNA sequence similarity is set at homology no less than 98% [23], our result has confirmed that strain D5 belongs to *Pseudomonas*.

### PCR Amplification Results of Homology Analysis of *nodA* Gene Fragment

*nodA* belongs to common nodulation genes, and is one of the key nodulation genes of Rhizobia. The presence or absence of *nodA* determines whether a plant can be nodulated. **Figure 4** shows the electrophoretogram of the PCR products of the 685 bp *nodA* fragment amplified from strain D5. We submitted the sequence to the GeneBank and obtained a sequence number as D5.sqn *nodA* JX162031. The phylogenetic tree showed that the *nodA* sequence of strain D5 was at the same branch as *B. elkanii* (AB610461), *B. elkanii* (HQ259488), *B. elkanii* (HQ259471), *B.* sp. (AJ430728), *B. elkanii* (AJ300251), *B. elkanii* (AB610460), *B. elkanii* (AM117554), *B. elkanii* (U04609) and *B. elkanii* (AB610459); the sequence homology between the *nodA* sequence of strain D5 and that of these bacteria was 95%, and the genetic distance was 0.01682 (**Figure 5**).

### PCR Amplification Results of Homology Analysis of *nifH* Gene Fragment

*nifH* is a structural gene of nitrogenase. The presence or absence of *nifH* determines whether a plant can fix nitrogen. **Figure 6** shows the electrophoretogram of the PCR products of the 740 bp *nifH* fragment amplified from strain D5. We submitted the sequence to the GeneBank and obtained a sequence number as D5.sqn *nifH* JX162030. As shown in the phylogenetic tree constructed with strain D5 *nifH* gene (**Figure 7**), strain D5 *nifH* gene and *B.* sp. (HQ259550) formed a branch; the homology between them was 98%, and the genetic distance was 0. The homology between this branch and another *B.* sp. DG (HM063961.1) was 99%, and the genetic distance was 0.007906.

## Discussion

Currently there are six genera of bacteria that can nodulate legumes, including *Rhizobium*, *Sinorhizobium*, *Allorhizobium*, *Mesorhizobium*, *Bradyrhizobium*, and *Azorhizobium* [24]. Strain D5 is isolated from the nodules of *A. confusa*, yet it does not belong to any of these six genera. Based on the Blast result of its complete 16S rRNA sequence and the phylogenetic tree constructed with the 16S rRNA sequence, strain D5 belongs to *Pseudomonas*. This study shows that under strict sterile conditions, strain D5 can successfully nodulate *A. confusa, A. crassicarpa* and *A. mangium*, and the nodules all have nitrogenase activity; *nodA* nodulation gene and *nifH* nitrogen-fixing gene are amplified from strain D5, suggesting that there is a diversity of endophytic nitrogen-fixing bacteria symbiotic with *A.* spp., and demonstrating there are strains in *Pseudomonas* that contain symbiotic nodulation gene and nitrogen-fixing gene. This has not been reported previously.

In the 1980’s, *P. stutzeri* A1501 was isolated from the root soil of Chinese southern rice; whole genome “shotgun” method was used to complete the whole genome sequencing of this bacterium, and the genome structure and function were analyzed [25]. That was the first non-legume symbiotic nitrogen-fixing bacterium whose whole genome sequencing analysis was ever completed in the world. Nitrogenase-related genes in *P. stutzeri* A1501 have been cloned [26]–[29], however its nodulation genes have not been studied.

*Bradyrhizobium* that shares 95% homology with the *nodA* gene and 98% homology with the *nifH* gene of strain D5 belonging to *Pseudomonas* is a slow-growing genus of rhizobia. A relatively high homology between certain specific gene of two bacterial strains that belong to different genera may be due to horizontal gene transfer (HGT) between bacteria. HGT can occur through transformation (bacteria absorbing free DNA), conjugation (between bacteria, mediated by plasmid) and transduction (between bacteria and virus). Rhizobia plasmids carry genes related to functions including nodulation and symbiotic nitrogen fixing, and are transferrable [30]. Studies have shown that rhizobia plasmids can be replicated and expressed in non-rhizobia including *P. aeruginosa* [31] and *Agrobacterium tumefaciens* [32]. Why is there the high homology between the *nodA* and *nifH* genes of strain D5 and those of *Bradyrhizobium*? One possible explanation could be that the symbiotic nodulation gene *nodA* and the nitrogen-fixing gene *nifH* carried by rhizobia plasmids are transferred between bacteria through conjugation, and that these genes are replicated and expressed in strain D5, equipping strain D5 with the ability to nodulate plants and fix nitrogen. To test this hypothesis further investigations on *Bradyrhizobium* plasmids and the mechanism of its conjugation with strain D5 are needed. This study sets foundation for future research, and provides scientific basis for investigations on HGT and gene recombination.

## Materials and Methods

### Strain

Strain D5 was isolated from nodules of *A. confusa*. The sampling location was at Chongzuo, Guangxi (21°36′ to 23°22′ N and 106°33′ to 108°6′ E). There is a wide stratigraphic distribution of limestone in this region. The sample was collected from a shallow and dry soil horizon.

The stored strain was cultured in a shaking flask containing YMB medium for 5∼7 d (28°C, 120 r/min) to be activated [14].

### Inoculation and Cross-inoculation Experiments

The inoculation experiments were performed under strict sterile conditions. The inoculated plants were *A. confusa, A. crassicarpa*, *A. mangium* and *Glycine max* (Linn.) Merr. To avoid infections caused by microorganisms in the soil, Acacia seeds were directly collected from the trees; they were then subject to surface sterilization twice as follows: soaked in 75% ethanol for 1–2 min, rinsed by sterile water for 4 times, soaked in 0.1% acidic mercuric chloride solution for 7–8 min, rinsed by sterile water for 6 times. Inoculation of strain D5 and seedling cultivation was carried out in fully enclosed settings. In each plant 2 ml logarithmic-phase culture of strain D5 was used for inoculation, and 10 plants for each species were used. Plants that were not inoculated or inoculated with other *Pseudomonas* strains were used as the control [33].

### Measurement of Nitrogenase Activity

The nodules of *A. confusa* are relatively small in size. In order to avoid loss of nitrogenase activity when removing the nodules from the plants, the intact root system containing the nodules was placed in a 10 ml small bottle, and acetylene reduction method was used to measure nitrogenase activity. Each measurement was repeated twice. Un-inoculated root systems were used as controls. The nitrogenase activity was calculated using the following formula [33]:

E (nmol C_2_H_4_·g^−1^ biomass·min^−1^) = K×ethylene peak height for sample (µV) × volume of reaction bulb (µL)/ethylene peak height for standard (µV) × loading volume of sample ethyne (µL) × biomass (g) × reaction time (min), where, K is a constant.

### Total DNA Extraction from the Strain

Bacterial genomic DNA extraction kit manufactured by Beijing Tiangen Bio Inc. was used for total DNA extraction. First 2 ml logarithmic-phase culture of strain D5 was centrifuged at 10000 rpm to collected precipitated biomass; DNA was extracted following the kit instruction and then subject to 1.2% agarose gel electrophoresis; the absorption values of the nucleic acids were used for concentration and purity measurements.

### PCR of 16S rRNA

The primers used were as follows: forward primer Pf (5′-AGA GTT TGA TCA TGG CTC AG-3′), and reverse primer Pr (5′-TAC GGT TAC CTT GTT ACG ACTT-3′).

The totals volume of PCR mixture was 50 µl, including: template DNA 2 µl, 10 µmol/L forward primer Pf and reverse primer Pr 2 µl each, 10×PCR buffer 5 µl, 2.5 mmol/L dNTP 4 µl, Taq polymerase 2U; dd H_2_O was added to make a total volume of 50 µl.

Reaction conditions: denaturation: 96°C 2 min, denaturation: 95°C 40 s, renaturation: 54°C 50 s, extension 72°C 1.5 min, 30 cycles, extension: 72°C 5 min.

### PCR of *nodA* Gene

Forward primer for *nodA* gene amplification was *nodA*-1(5′-TGC RGT GGA ARN TRN NCT GGG AAA-3′), and the reverse primer was *nodA*-2(5′-GGN CCG TCR TCR AAW GTC ARG TA-3′).

The totals volume of PCR mixture was 25 µl, including: template DNA 2 µl, 10 µmol/L forward primer *nodA*-1 and reverse primer *nodA*-2 1 µl each, 10×PCR buffer 2.5 µl, 2.5 mmol/L dNTP 2 µl, Taq polymerase 1U; dd H_2_O was added to make a total volume of 25 µl.

Reaction conditions: denaturation: 94°C 5 min, denaturation: 94°C 30 s, annealing: 50°C 45 s, extension 72°C 60 s, 34 cycles, extension: 72°C 5 min.

### PCR of *nifH* Gene

Forward primer for *nifH* gene was *nifH*-f (5′-AAA GGY GGW ATC GGY AAR TCC ACC AC-3′), and the reverse primer was *nifH*-r (5′-TTG TTS GCS GCR TAC ATS GCC ATC AT-3′).

The totals volume of PCR mixture was 25 µl, including: template DNA 2 µl, 10 µmol/L forward primer *nifH*-f and reverse primer *nifH*-r 1 µl each, 10×PCR buffer 2.5 µl, 2.5 mmol/L dNTP 2 µl, Taq polymerase 1U; dd H_2_O was added to make a total volume of 25 µl.

Reaction conditions: denaturation: 94°C 3 min, denaturation: 94°C 45 s, renaturation: 65°C 45 s, extension 72°C 2 min, 33 cycles, extension: 72°C 7 min.

### Examination of PCR Products

After PCR reaction was over, 2.5 µl amplification sample was loaded to 1.2% agarose gel for electrophoresis at 100 V. Amplification samples showing bright, pure, specific electrophoretic bands were sent to Beijing Sainuo Biotech Service Inc. for sequencing.

### Gene Sequence Analysis

We logged into NCBI to access GenBank for sequence similarity analysis and homology comparison of the obtained 16S rRNA, *nodA* and *nifH* gene sequences. We then used CLUSTALX for multiple sequence comparison, and MEGA4.0 software to construct the phylogenetic trees based on 16S rRNA, *nodA* or *nifH* gene sequence. Reference strains and their accession numbers used for sequence similarity and homology comparisons were all from GenBank.
